# Quality and Safety of Meat Products

**DOI:** 10.3390/foods7080118

**Published:** 2018-07-26

**Authors:** Begoña Panea, Guillermo Ripoll

**Affiliations:** Centro de Investigación y Tecnología Agroalimentaria de Aragón, 50059 Zaragoza, Spain; gripoll@aragon.es

The Rome Declaration on World Food Security includes the right of everyone to have access to safe and nutritious food, and the World Summit on Food recognized the link between food safety and quality. In 2020, the world’s population will surpass 7.5 billion of people and the main increases will be in developing countries. It is well known that development stimulates an increase in the demand for high-quality protein, and among candidates, food undoubtedly includes meat and meat products. Thus, this increase in population poses great food challenges in general, and to the meat industry in particular. To meet the needs of these populations, we will have to reorganize production systems and the distribution of food and some of the changes will bring potential problems to food safety and nutritional quality. Food-borne diseases are a major problem around the world, both in regards to human suffering and with respect to economic costs. Scientific advances have allowed to better know the nutritional characteristics of foods and their effects on health. This means that a large proportion of consumers are much more conscious with respect to what they eat, and their demands for quality food. Food quality is a complex term that includes, in addition to safety, other intrinsic characteristics, such as appearance, color, texture and flavor and also extrinsic characteristics, such as labelling or certification. Scientists have much to contribute to this new scenario. Our role will be critical to ensure future population health, nutrition and sensory-acceptable foods.

The current Special Issue collected some interesting papers concerning several aspects of meat quality and safety.

Firstly, this special issue contained a very interesting review entitled “Carbon Monoxide in Meat and Fish Packaging: Advantages and Limits” [1]. This review was undertaken to present the most comprehensive and current overview of the widely-available, scattered information about the use of CO in the preservation of muscle foods. The advantages of CO and its industrial limits are presented and discussed. The most recent literature on the consumer safety issues related to the use of CO and consumer acceptance of CO especially in meat packaging systems were also discussed.

Regarding safety matters, special issue enclosed an experimental work and a review. *Escherichia coli O157:H7* is one of the main safety problems in the meat industry since it generates toxins that can cause severe hemolytic uremic syndrome in infected humans. Then, paper entitled “A Comparison Study of Quality Attributes of Ground Beef and Veal Patties and Thermal Inactivation of Escherichia coli O157:H7 after Double Pan-Broiling Under Dynamic Conditions” [2] aimed to investigate the quality variances, including color variation in non-intact coarse ground beef and veal patties during aerobic storage and cooking and to evaluate the thermal inactivation of *E. coli O157:H7* in coarse ground beef and veal patties. Thermal processing was widely used to inactivate spoilage and foodborne pathogens. The novelty of this study is that the thermal kinetics study was conducted in commercial-sized patties cooked on a griller instead of using small amounts of meat heated in a water bath. 

Other important pathogens in meat and meat products, such as *Salmonella* spp., Listeria monocytogenes, *Staphylococcus aureus* and *Campylobacter* spp. are considered in the review entitled Prevalence of Pathogens in Poultry Meat: A Meta-Analysis of European Published Surveys [3]. This review summarized the levels of incidence of *Salmonella* spp., Listeria monocytogenes, *Staphylococcus aureus* and *Campylobacter* spp. in poultry meat commercialized in Europe. Incidence data and study characteristics were extracted from 78 studies conducted in 21 European countries. The results of this meta-analysis highlight that further risk management strategies are needed to reduce pathogen incidence in poultry meat throughout the entire food chain across Europe, in particular for *S. aureus* and *Campylobacter* spp.

Concerning meat quality, several problems and tendencies of the meat industry was approached. New marketing possibilities, development of new products, new conservation methodologies, the development of meat flavors depending on storage conditions and the impact of cooking methods on human health was studied. 

In many Mediterranean countries, the ovine meat sector is important in the maintenance and sustainability of rural areas, but ovine meat has, in general, low prices, especially meat from cull ewes. Then elaboration of meat-based products could also have a positive economic outcome for breeders. In this sense, two papers proposed two different strategies. First, paper entitled “Consumer Acceptability of Dry Cured Meat from Cull Ewes Reared with Different Linseed Supplementation Levels and Feeding Durations” [4] examined the influence of the different levels of linseed supplementation and feeding duration in cull ewes on the consumer acceptability of the dry cured sheep meat ‘cecina’, a product particularly appreciated and consumed in some countries of the Mediterranean area. 

Also considered how to do with meat from old animals which usually have low market prices, we found the paper entitled “Improving Cull Cow Meat Quality Using Vacuum Impregnation” [5]. At this time, the proposal is to enhance meat quality from cull cow throughout varying the pressure and time of impregnation with an isotonic solution of sodium chloride. Paper studied the microstructural changes associated to the treatment and concluded that vacuum impregnation has a considerable influence on its tenderness and juiciness after cooking and that it may present a good alternative in the production of moisture-enhanced meat products, offering similar weight gain as that of traditional injected meat but with lower levels of sodium and a more complete distribution of the brine into meat.

Paper entitled “Consumer Perception of the Quality of Lamb and Lamb Confit” [6] started from the assumption that the patterns of food consumption are constantly changing and that these changes are due both to socioeconomic and cultural trends and to the specific lifestyles of consumer groups. Then, the paper aimed to identify the profiles of light lamb meat consumers according to their orientation toward convenience, to characterize these profiles according to their socioeconomic characteristics and their preferences regarding the intrinsic and extrinsic quality cues of light lamb meat and to analyze the willingness to pay for light lamb confit.

In Volatile Profile of Raw Lamb Meat Stored at 4 ± 1 °C: The Potential of Specific Aldehyde Ratios as Indicators of Lamb Meat Quality paper [7], aldehyde ratios are correlated to study shelf life and degree of oxidation test data and proposed as markers of lamb meat freshness and overall quality. Results of the present study showed that the evolution of hexanal, heptanal, and nonanal during storage time could be proposed as overall indicators of lamb meat freshness and degree of oxidation, since they were strongly correlated with shelf life and TBA test data. In addition, a hexanal to nonanal ratio equal or lower than 2.45 was proposed as an indicator of lamb meat freshness and overall quality.

New products and market tendencies are studied in Meat Quality Derived from High Inclusion of a Micro-Alga or Insect Meal as an Alternative Protein Source in Poultry Diets: A Pilot Study [8]. This study investigates the possibility of replacing soybean diets by two proteins that could be produced outside the arable farming system, and it focused on one micro-algae source (Spirulina, *Arthrospira platensis*) and one insect protein source (black soldier fly, *Hermetia illucens* L.). The study was conducted in poultry and concluded that based on their nominal effects on meat quality, Spirulina and Hermetia larval meal remain two potential protein alternatives for poultry diets.

Finally, paper entitled “Effect of Par Frying on Composition and Texture of Breaded and Battered Catfish” [9] aimed to proposed healthier alternatives to prepare a very popular meal, such as fried catfish. The overall goal of this study was to make a battered catfish product that could be baked and have a lower percentage of oil-based calories than equivalent par-fried products. The study examined the effect of different batters (rice, corn, and wheat) and the effect of par frying on the composition and texture properties of baked catfish. Some significant differences were found between treatments and, as a general conclusion, it showed that the texture of the coatings in the par-fried treatments were significantly greater for hardness attributes.
